# Individual and combined contributions of non-high-density lipoprotein cholesterol and brachial-ankle pulse wave velocity to cardiovascular disease risk: Results of a prospective study using the Kailuan cohort

**DOI:** 10.3389/fcvm.2023.1105464

**Published:** 2023-02-09

**Authors:** Qiongbing Zheng, Hui Wang, Xianxuan Wang, Youmian Lan, Weiqiang Wu, Xinran Yu, Zegui Huang, Zekai Chen, Zefeng Cai, Qi Lin, Houshi Zhou, Yongdong Zhu, Muyuan Liu, Kuangyi Wu, Huancong Zheng, Shouling Wu, Youren Chen

**Affiliations:** ^1^Department of Neurology, Shantou Central Hospital, Shantou, China; ^2^School of Nursing, Shandong First Medical University and Shandong Academy of Medical Sciences, Jinan, China; ^3^Graduate School, Shantou University Medical College, Shantou, Guangdong, China; ^4^Department of Cardiology, Second Affiliated Hospital of Shantou University Medical College, Shantou, Guangdong, China; ^5^Graduate School, North China University of Science and Technology, Tangshan, China; ^6^Department of Epidemiology, University Medical Center Groningen, University of Groningen, Groningen, Netherlands; ^7^Department of Head and Neck, Cancer Hospital of Shantou University Medical College, Shantou, China; ^8^Department of Cardiology, Kailuan General Hospital, Tangshan, China

**Keywords:** non-high-density lipoprotein cholesterol, brachial-ankle pulse wave velocity, cardiovascular diseases, Kailuan cohort, combined effect

## Abstract

**Objective:**

We aimed to characterize the relationship of a combination of circulating non-high-density lipoprotein-cholesterol (non-HDL-C) concentration and brachial-ankle pulse wave velocity (baPWV) with cardiovascular disease (CVD).

**Methods:**

We performed a prospective cohort study of the residents of the Kailuan community, with data from a total of 45,051 participants being included in the final analysis. The participants were allocated to four groups according to their non-HDL-C and baPWV status, each of which was categorized as high or normal. Cox proportional hazards models were used to explore the relationships of non-HDL-C and baPWV, individually and in combination, with the incidence of CVD.

**Results:**

During the 5.04-year follow-up period, 830 participants developed CVD. Compared with the Normal non-HDL-C group independently, the multivariable adjusted hazard ratios (HRs) and 95% confidence intervals (CIs) for CVD in the High non-HDL-C was 1.25 (1.08–1.46). Compared with the Normal baPWV group independently, the HRs and 95% CIs for CVD in the High baPWV was 1.51 (1.29–1.76). In addition, compared with the Normal both non-HDL-C and baPWV group, the HRs and 95% CIs for CVD in the High non-HDL-C and normal baPWV, Normal non-HDL-C and high baPWV, and High both non-HDL-C and baPWV groups were 1.40 (1.07–1.82), 1.56 (1.30–1.88), and 1.89 (1.53–2.35), respectively.

**Conclusion:**

High non-HDL-C concentration and high baPWV are independently associated with a higher risk of CVD, and individuals with high both non-HDL-C and baPWV are at a still higher risk of CVD.

## 1. Introduction

Arteriosclerosis is not only a sign of advanced vascular aging, but also a potent, independent risk factor for cardiovascular disease (CVD), renal failure, cognitive dysfunction, and all-cause mortality (1–4). There is a linear correlation between the severity of arteriosclerosis and CVD, and the Framingham study showed that each standard deviation (SD) increment in carotid-femoral artery pulse wave velocity (cfPWV) is associated with a 48% higher risk of cardiovascular disease (5, 6). Moreover, a meta-analysis of aortic pulse wave velocity (PWV) showed that an increase in aortic PWV of 1 m/s is associated with age-, sex-, and other risk factor-adjusted increases of 15, 14, and 15% in the risks of all-cause mortality, and total CVD morbidity and mortality, respectively (7).

In 2001, the National Cholesterol Education Program of the United States first proposed the utility of measuring the circulating concentration of non-high-density lipoprotein-cholesterol (non-HDL-C), which includes multiple types of lipoprotein and cholesterol that cause atherosclerosis, such as low-density lipoprotein-cholesterol (LDL-C) (8). However, no matter whether it is used to predict the morbidity or mortality associated with CVD or to evaluate the efficacy of lipid-lowering therapy, non-HDL-C has been shown to superior to LDL-C (9–11). Mora et al. (12) reported that female patients with LDL-C concentrations below the median value and non-HDL-C concentrations above the median have a threefold higher risk of CVD than those with non-HDL-C concentrations below the median value.

Atherosclerosis, as well as non-HDL-C specifically, is a risk factor for cardiovascular events. Several previous studies have shown a close correlation between non-HDL-C concentration and brachial-ankle pulse wave velocity (baPWV) (13–15). Many previous studies (16–18) have shown the correlation between non-HDL-C or baPWV with CVD separately, but few studies about the relationship of a combination of both non-HDL-C and baPWV with CVD. Therefore, to remedy this deficiency, we evaluated the relationship of non-HDL-C or baPWV with CVD separately, which was similar as previous studies. After that, we investigated the relationship of a combination of both non-HDL-C and baPWV with CVD. We used data from the Kailuan Study (Registration Number: CHICTR-TNRC-11001489) to explore the relationships of non-HDL-C and baPWV, individually and in combination, with CVD events.

## 2. Materials and methods

### 2.1. Study participants

The Kailuan study (registration number: ChiCTR-TNRC-11001489) is an ongoing community-based study of the risk factors and interventions for CV and related diseases that commenced in 2006. According to standardized uniform design, face-to-face questionnaire interviews (demographic characteristics, disease history, lifestyles, etc.), physical examinations (body weight, height, waist circumference, blood pressure, etc.), and laboratory tests (fasting blood glucose, lipids profile, etc.) were conducted by trained physicians or nurses in every circle for 2 years, with a total of six being completed to date. In addition to the usual measurements performed on these occasions, including of blood lipid concentrations, baPWV measurement was commenced for some of the participants in 2010–2011. For the present study, individuals who participated in the follow-up examinations during 2010–2011, 2012–2013, 2014–2015, 2016–2017, and 2018–2019, and who underwent baPWV measurements, were studied. The study conformed with the Declaration of Helsinki and was approved by the Ethics Committee of Kailuan General Hospital (approval number: 2006-05). All of the participants provided their written informed consent.

### 2.2. Inclusion and exclusion criteria

#### 2.2.1. Inclusion criteria

① Individuals who participated in follow-up examinations during 2010/11, 2012/13, 2014/15, 2016/17, and 2017/18. ② Those who underwent baPWV measurements at each of these follow-up examinations. ③ Those who agreed to participate in the present study and provided their written informed consent.

#### 2.2.2. Exclusion criteria

**Figure 1:**
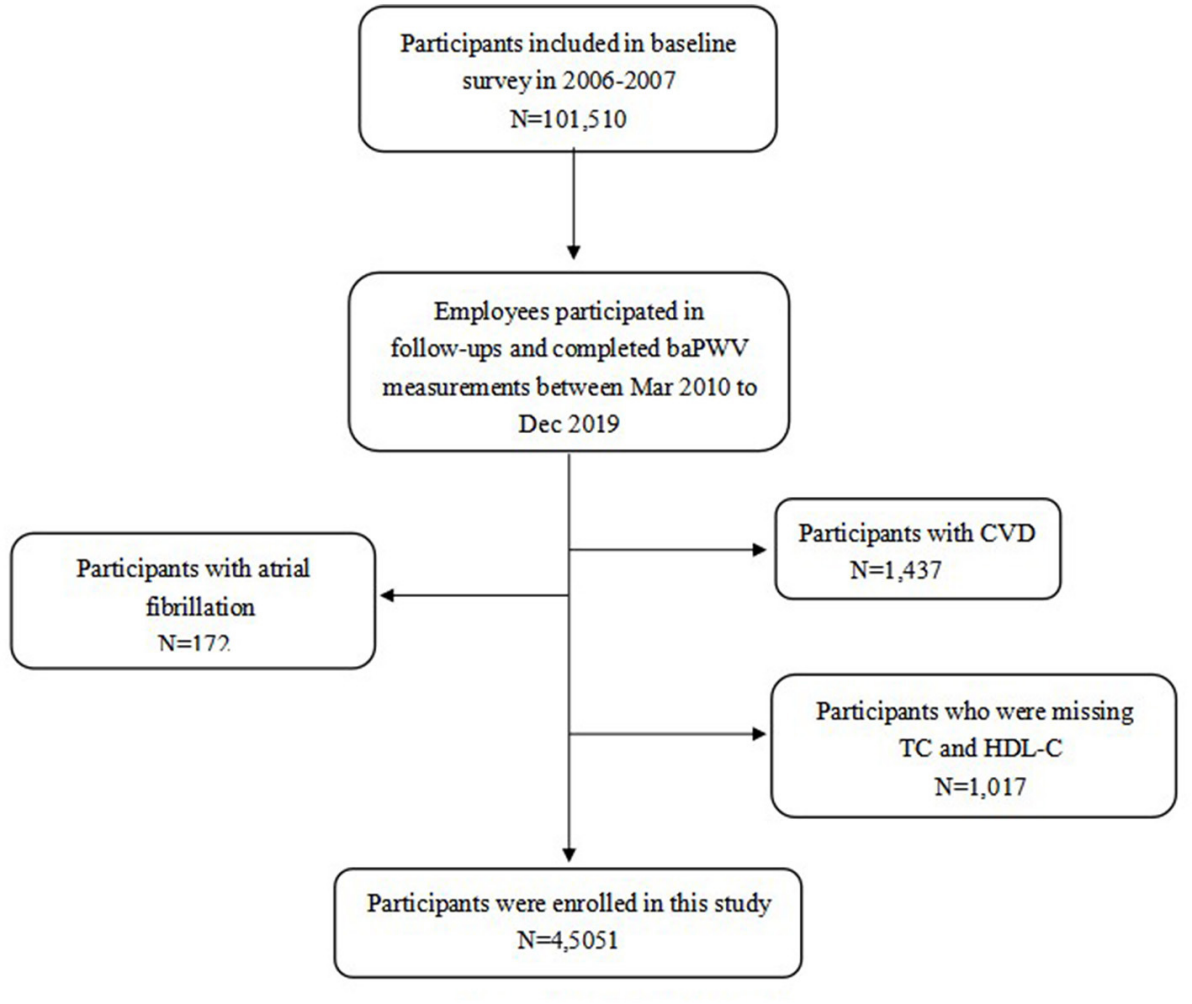
Flowchart of this study.

① Participants with a previous history of CVD. ② Those with electrocardiographic data suggestive of atrial fibrillation at the time of baPWV testing. ③ Those with missing data for circulating total cholesterol (TC), and HDL-C concentrations, as shown in Figure 1.

### 2.3. Data collection

#### 2.3.1. Anthropometric parameters and related definitions

A standardized questionnaire was completed by members of the Kailuan study cohort at each follow-up visit to collect information regarding age, sex, lifestyle (smoking status, alcohol consumption status, and educational level), medical history, and the use of medication. Measurements of height, body mass, and blood pressure were performed by a professional according to a standard protocol and to 0.1 cm for height and 0.1 kg for body mass. A calibrated mercury column sphygmomanometer was used to measure the right brachial artery blood pressure, and three consecutive measurements were made at intervals of 1–2 min, and if the difference between the measurements exceeded 5 mmHg, a further three measurements were made. The mean of the three measurements was used in analyses. Body mass index (BMI) was calculated as body mass (kg)/height^2^ (m^2^). Physical activity was defined as aerobic exercise ≥3 times/week for ≥30 min on each occasion. A smoker was defined as someone who smoked a mean of at least one cigarette per day during the preceding year, and an alcohol consumer was defined as someone who drank a mean of 100 mL of liquor (50% alcohol or more) per day for at least 1 year. Hypertension was defined using a systolic blood pressure (SBP) ≥140 mmHg and/or a diastolic blood pressure (DBP) ≥90 mmHg, the use of anti-hypertensive drugs, or a self-reported history of hypertension (19). Diabetes mellitus was defined using a fasting blood glucose (FBG) ≥7 mmol/L, the use of hypoglycemic drugs, or a self-reported history of diabetes mellitus (20).

#### 2.3.2. Biochemical index test

After fasting for at least 8 h, 5 mL of fasting elbow venous blood were drawn between 07:00 and 09:00 on the day of physical examination for the measurement of the TC, HDL-C, LDL-C, TG, Hs-CRP, and fasting blood glucose (FBG) concentrations. The serum concentrations of FBG, LDL-C, and HDL-C were measured by the hexokinase/glucose-6-phosphate dehydrogenase method, and direct method-surfactant clearance method, respectively, using a Hitachi 7600 autoanalyzer (Hitachi, Tokyo, Japan) at the central laboratory of Kailuan General Hospital.

Non-HDL-C concentration was calculated as the sum of the concentrations of lipoprotein-cholesterol other than HDL-C using non-HDL-C = TC – HDL-C.

#### 2.3.3. baPWV testing

baPWV testing was performed on the day of follow-up examination between 07:00 and 09:00. The room temperature of the examination room was maintained at 22–25°C and measurements were made by trained professionals. A BP-203RPEIII networked atherosclerosis detection device [Omron Health Medical (China) Co., Ltd., Beijing, China] was used to measure baPWV, and the blood pressure values of the extremities were also recorded. The participants were asked not to smoke and to rest for more than 5 min prior to the measurements, and they remained supine during the test. The measurement was repeated twice for each participant and the second set of values obtained were used. The larger baPWV value for the left and right sides was used in the analyses.

### 2.4. Non-HDL-C and baPWV status

According to the 2016 Chinese Guidelines for the Management of Dyslipidemia in Adults (21), non-HDL status was classified as normal (<4.9 mmol/L, without the use of lipid-lowering drugs), or high (≥4.9 mmol/L or the use of lipid-lowering drugs). As in a previous study (22), we used age- and sex-specific cut-off values to categorize participants according to baPWV, because these two factors, age and sex, are key correlates of baPWV. Therefore, high baPWV was defined using a baPWV of less than the ≥5-year age- and sex-specific median value.

To determine the combined effects of high non-HDL-C and high baPWV, the participants were placed into four groups: Normal both non-HDL-C and baPWV, High non-HDL-C and normal baPWV, Normal non-HDL-C and high baPWV, and High both non-HDL-C and baPWV groups.

### 2.5. Outcomes

The start of the follow-up period was defined as the time point at which the participant underwent their first baPWV measurement and the end was defined as the time point when the individual participated in the 2018–2019 follow-up examination. The occurrence of a CVD was used as the endpoint.

The CVD outcomes were defined as myocardial infarction (MI) or ischemic stroke. We used ICD-10th revision codes to identify CVD cases I21 for MI, and I63 for ischemic stroke (23). The databases of CVD diagnoses were obtained from the Municipal Social Insurance Institution and the Hospital Discharge Register. These were updated annually during the follow-up period. An expert panel collected and reviewed the annual discharge records from the 11 participating hospitals to identify patients who were suspected of having CVD. A diagnosis of MI was made on the basis of the symptoms, electrocardiographic findings, and changes in myocardial enzyme activities, according to the World Health Organization's Multinational Monitoring of Trends and Determinants in Cardiovascular Disease criteria (24). Stroke was diagnosed on the basis of neurological signs, symptoms, and the findings of neuroimaging, including computed tomography and magnetic resonance imaging, according to the World Health Organization's criteria (25). For those who experienced two or more events, the time and nature of the first event was recorded as the endpoint, and those who did not experience an event underwent their last follow-up examination on December 31, 2019.

### 2.6. Statistical analysis

The baseline information used in this study was the data collected during the physical examination performed at the time of baPWV measurement. Normally distributed continuous data are expressed as mean ± standard deviation and one-way ANOVA was used for comparisons between multiple groups. Non-normally distributed continuous data were expressed as median (25th and 75th percentiles) and the Wilcoxon rank sum test was used for comparisons between groups. Categorical data were expressed as number of cases (%) and the chi-square test was used for comparisons between groups. Differences of basic characteristics between four groups were compared with Bonferoni correction.

The cumulative incidence of CVD for each group was calculated using the Kaplan-Meier method and they were compared using the log-rank test. First, multivariable Cox proportional hazards models were used to evaluate the individual relationships between non-HDL-C or baPWV and the incidence of CVD and to evaluate the relationships between the four groups and the incidence of CVD. We adjusted for age (continuous variable), sex, BMI (continuous variable), MAP (continuous variable), Hs-CRP concentration (continuous variable), LDL-C (continuous variable), smoking status (categorical variable, yes or no), alcohol consumption status (categorical variable, yes or no), physical activity (categorical variable, yes or no), educational level (categorical variable, yes or no), family history of diabetes (categorical variable, yes or no), and the use of anti-hypertensive agents (categorical variable, yes or no) and lipid-lowering drugs (categorical variable, yes or no) in the multivariable models. Covariates were selected on the basis of previous reports of their associations with CVD (26, 27).

The data were also analyzed after stratification according to age, sex, and whether or not to use lipid-lowering drugs. To test the robustness of the results, the following sensitivity analyses were performed: (1) the exclusion of individuals who developed CVD outcomes occurring within the first year of the study (*n* = 313); (2) the exclusion of individuals with ABI ≤ 0.9 (*n* = 967); (3) after the exclusion of participants who underwent treatment with anti-hypertensive, hypoglycemic, or lipid-lowering medications (*n* = 13,288). The data were analyzed using SAS v.9.4 (Cary, NC, USA) and differences were considered statistically significant when *P* < 0.05 (two-sided).

## 3. Results

### 3.1. Characteristics of the study participants

The mean age of the 45,051 participants was 49.21 ± 13.00 years. The mean serum non-HDL-C concentration was 3.60 ± 0.99 mmol/l and the mean baPWV was 1,513.5 ± 348.7 cm/s. The Normal both non-HDL-C and baPWV, High non-HDL-C and normal baPWV, Normal non-HDL-C and high baPWV, and High both non-HDL-C and baPWV groups contained 17,510, 4,784, 16,278, and 6,479 participants, respectively. Compared with Normal both non-HDL-C concentration and baPWV, those with High both non-HDL-C and baPWV were older; had higher SBP, DBP, BMI, and baPWV; had higher triglyceride (TG), TC, hs-CRP, and FBG concentrations; and had the highest prevalences of hypertension, diabetes, smoking, and the use of anti-hypertensive drugs (*P* < 0.01) (all *P* < 0.01; Table 1).

**Table 1:** Baseline characteristics of participants by non-HDL-C and baPWV status.

	**All** **(*N* = 45,051)**	**Normal both non-HDL-C and baPWV** **(*N* = 17,510)**	**High non-HDL-C and normal baPWV** **(*N* = 4,784)**	**Normal non-HDL-C and high baPWV** **(*N* = 16,278)**	**High both non-HDL-C and baPWV** **(*N* = 6,479)**	***P-*value**
Age (years)	49.21 ± 13.00	49.44 ± 13.06	49.10 ± 12.15	49.15 ± 13.54	48.79 ± 12.00^a^	0.05
BMI (kg/m^2^)	25.03 ± 3.35	24.39 ± 3.18	26.52 ± 3.12^a^	24.67 ± 3.32^a^^b^	26.58 ± 3.23^a^^c^	< 0.01
SBP (mmHg)	131.78 ± 19.01	125.87 ± 17.14	130.99 ± 16.64^a^	135.20 ± 19.86^a^^b^^c^	139.74 ± 18.31^a^^b^^c^	< 0.01
DBP (mmHg)	81.94 ± 10.94	78.80 ± 10.07	82.14 ± 10.05^a^	83.31 ± 11.08^a^^b^	86.88 ± 10.91^a^^b^^c^	< 0.01
MAP (mmHg)	98.56 ± 12.49	94.49 ± 11.33	98.42 ± 11.05^a^	100.61 ± 12.78^a^^b^	104.50 ± 12.14^a^^b^^c^	< 0.01
baPWV (cm/s)	1,513.47 ± 348.73	1,316.49 ± 198.20	1,343.64 ± 171.82^a^	1,686.17 ± 373.18^a^^b^	1,737.34 ± 341.45^a^^b^^c^	< 0.01
TG (mmol/l)	1.28 (0.87–1.96)	1.02 (0.75–1.36)	2.62 (2.09–3.54)^a^	1.10 (0.81–1.46)^a^^b^	2.81 (2.22–3.86)^a^^b^^c^	< 0.01
TC (mmol/l)	5.03 ± 1.02	4.90 ± 0.95	5.20 ± 1.07^a^	5.00 ± 0.99^a^^b^	5.33 ± 1.14^a^^b^^c^	< 0.01
HDL-C (mmol/l)	1.43 ± 0.36	1.51 ± 0.34	1.14 ± 0.27^a^	1.52 ± 0.35^b^	1.19 ± 0.29^a^^b^^c^	< 0.01
LDL-C (mmol/l)	2.88 ± 6.03	2.87 ± 8.74	2.84 ± 0.88	2.86 ± 1.37	2.97 ± 6.43	0.57
FBG (mmol/l)	5.78 ± 1.78	5.44 ± 1.29	5.90 ± 1.85^a^	5.84 ± 1.84^a^^b^	6.44 ± 2.42^a^^b^^c^	< 0.01
non-HDL-C (mmol/l)	3.60 ± 0.99	3.39 ± 0.90	4.06 ± 1.01^a^	3.48 ± 0.93^a^^b^	4.14 ± 1.07^a^^b^^c^	< 0.01
Hs-CRP (mg/l)	1.29 (0.51–3.00)	1.10 (0.48–2.70)	1.60 (0.70–3.41)^a^	1.20 (0.50–2.92)^a^^b^	1.69 (0.74–3.68)^a^^b^^c^	< 0.01
Diabetes mellitus, *n* (%)	6,168 (13.69)	1,384 (7.90)	745 (15.57)^a^	2,462 (15.12)^a^	1,577 (24.34)^a^^b^^c^	< 0.01
Hypertension, *n* (%)	25,216 (55.97)	7,912 (45.19)	2,584 (54.01)^a^	10,101 (62.05)^a^^b^	4,619 (71.29)^a^^b^^c^	< 0.01
Anti-hypertension drugs, *n* (%)	10,537 (23.39)	2,545 (14.53)	1,047 (21.89)^a^	4,562 (28.03)^a^^b^	2,383 (36.78)^a^^b^^c^	< 0.01
Lipid-lowering drugs, *n* (%)	2,927 (6.50)	802 (4.58)	500 (10.45)^a^	895 (5.50)^a^^b^^c^	730 (11.27)^a^^c^	< 0.01
Smoking, *n* (%)	16,241 (36.05)	5,897 (33.68)	2,097 (43.83)^a^	5,479 (33.66)^b^	2,768 (42.72)^a^^c^	< 0.01
Drinking, *n* (%)	14,277 (31.69)	5,162 (29.48)	1,810 (37.83)^a^	4,802 (29.50)^b^	2,503 (38.63)^a^^c^	< 0.01
Exercise, *n* (%)	8,319 (18.47)	3,339 (19.07)	915 (19.13)	2,880 (17.69)^a^	1,185 (18.29)	< 0.01
Education > high school, *n* (%)	7,743 (17.19)	3,345 (19.10)	921 (19.25)	2,574 (15.81)^a^^b^	903 (13.94)^a^^b^^c^	< 0.01

P, comparison of baseline characteristics between different non-HDL-C and baPWV groups.

BMI, body mass index; DBP, diastolic blood pressure; FBG, fasting blood glucose; HDL-C, high-density lipoprotein cholesterol; hs-CRP, high-sensitivity C reactive protein; LDL-C, low-density lipoprotein cholesterol; PP, pulse pressure; SBP, systolic blood pressure; TC, total cholesterol; TG, triglyceride; non-HDL-C: non-high-density lipoprotein cholesterol.

a P < 0.05, compare with Normal both non-HDL-C and baPWV group.

b P < 0.05, compare with High non-HDL-C and Normal baPWV group.

c P < 0.05, compare with Normal non-HDL-C and high baPWV group.

### 3.2. Cumulative incidence of CVD in the various groups

**Figure 2:**
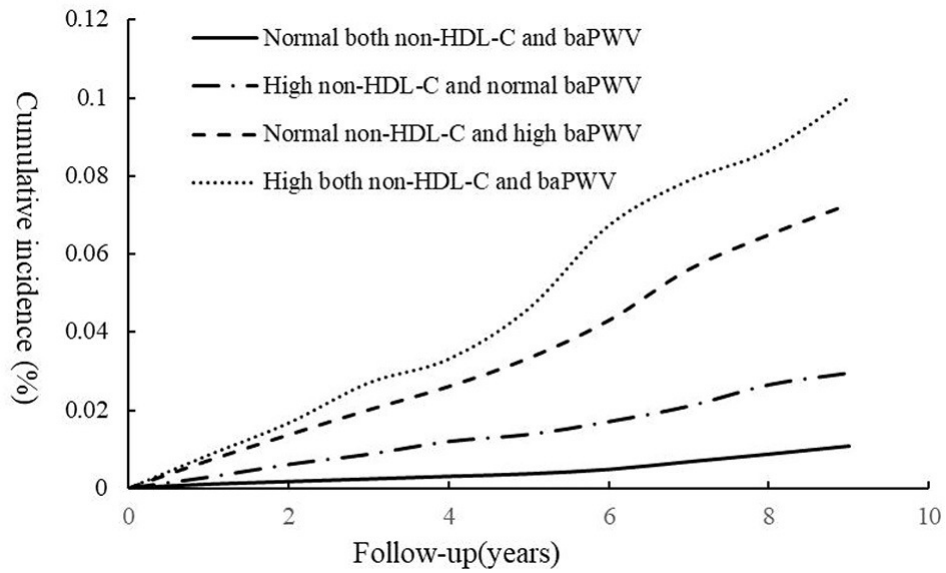
Kaplan–Meier incidence of CVD in groups with combination of normal or high non-HDL-C and baPWV. *p* < 0.05; log-rank test.

During a follow-up period of 5.04 ± 2.80 years, the cumulative incidences of CVD in the groups with High non-HDL-C and normal baPWV, Normal non-HDL-C and high baPWV, and High both non-HDL-C and baPWV showed a trend to increase vs. the group with normal non-HDL-C and normal baPWV (cumulative incidences of CVD for each group: 1.09, 2.96, 7.29, and 10.01; incidence densities for each group: 2.11/1,000, 3.59/1,000, 4.44/1,000, and 6.06/1,000 person-years; respectively); and the differences in the cumulative incidences of the endpoint events among the groups were statistically significant (log-rank test; all *P* < 0.05); as shown in Figure 2.

### 3.3. Relationships of high non-HDL-C concentration and high baPWV alone with the risk of CVD

The results of multivariate Cox regression analysis, with the incidence of CVD as the dependent variable and the baPWV (Normal baPWV group as the control group) and non-HDL-C (Normal non-HDL-C group as the control group) subgroups as the independent variables, showed that compared with the Normal non-HDL-C group, and the HR for cardiovascular events was 1.15 (1.07–1.24) per standard deviation increase in non-HDL-C concentration. In addition, the HR for CVD was 1.51 (1.29–1.76) for the High baPWV group vs. the Normal baPWV group, and the HR for CVD was 1.22 (1.14–1.31) per standard deviation increase in baPWV, as shown in Table 2.

**Table 2:** Influence of serum non-HDL-C or baPWV on CVD.

	**Case/total**	**Incidence (10 K/person-years)**	**Model 1**	**Model 2**	**Model 3**
**Non-HDL-C (mmol/l)**					
Normal HDL-C	560/33,788	3.22	1.00	1.00	1.00
High non-HDL-C	270/11,263	5.02	1.66 (1.43, 1.92)	1.28 (1.10, 1.49)	1.25 (1.08, 1.46)
Non-HDL-C _SD (0.99)			1.22 (1.15, 1.30)	1.15 (1.07, 1.25)	1.15 (1.07, 1.24)
**baPWV (cm/s)**					
Normal baPWV	272/22,294	2.40	1.00	1.00	1.00
High baPWV	558/22,757	4.89	2.15 (1.86, 2.48)	1.51 (1.29, 1.76)	1.51 (1.29, 1.76)
baPWV_SD (348.72)			1.43 (1.35, 1.52)	1.22 (1.15, 1.31)	1.22 (1.14, 1.31)

Model 1: adjusted for age, sex.

Model 2: included variables in model 1 and further BMI, MAP, Hs-CRP, LDL-C, Diabetes mellitus, physical activity, educational level, smoking, drinking, antihypertensive agents and lipid-lowering drugs therapy.

Model 3: included variables in model 2 and baPWV or non-HDL-C.

Non-HDL-C, non-high-density lipoprotein cholesterol; baPWV, brachial-ankle pulse wave velocity; CVD, cardiovascular disease; BMI, body mass index; TG, triglyceride; MAP, mean arterial pressure; Hs-CRP, high-sensitivity C reactive protein.

### 3.4. Relationships of non-HDL-C concentration and baPWV with the risk of CVD

Multivariate Cox regression analysis, with the occurrence of a CVD as the dependent variable and the various non-HDL-C and baPWV subgroups (Normal both non-HDL-C and baPWV group as the control group) as the independent variables, showed that compared with the Normal both non-HDL-C and baPWV group, the HRs for s in each group were 1.40 (1.07–1.82), 1.56 (1.30–1.88), and 1.89 (1.53–2.35), respectively (Table 3).

**Table 3:** Influence of combined effects of non-HDL-C and baPWV on CVD.

**Group**	**Case/total**	**Incidence (10 K/person-years)**	**Model 1**	**Model 2**
**Cardiovascular disease**				
Normal both non-HDL-C and baPWV	191/17,510	2.11	1	1
High non-HDL-C and normal baPWV	81/4,784	3.59	1.77 (1.36, 2.30)	1.40 (1.07, 1.82)
Normal non-HDL-C and high baPWV	369/16,278	4.44	2.20 (1.85, 2.62)	1.56 (1.30, 1.88)
High both non-HDL-C and baPWV	189/6,479	6.06	3.23 (2.64, 3.96)	1.89 (1.53, 2.35)
**Myocardial infarction**				
Normal both non-HDL-C and baPWV	39/17,510	0.52	1	1
High non-HDL-C and normal baPWV	24/4,784	1.32	2.59 (1.55, 4.31)	2.15 (1.27, 3.61)
Normal non-HDL-C and high baPWV	65/16,278	0.95	1.90 (1.28, 2.83)	1.47 (0.97, 2.22)
High both non-HDL-C and baPWV	39/6,479	1.53	3.25 (2.08, 5.09)	2.32 (1.32, 3.45)
**Ischemic stroke**				
Normal both non-HDL-C and baPWV	153/17,510	2.07	1	1
High non-HDL-C and normal baPWV	59/4,784	3.27	1.64 (1.22, 2.22)	1.27 (0.94, 1.73)
Normal non-HDL-C and high baPWV	306/16,278	4.52	2.30 (1.89, 2.79)	1.57 (1.28, 1.92)
High both non-HDL-C and baPWV	152/6,479	6.04	3.29 (2.63, 4.13)	1.84 (1.44, 2.34)

Model 1: adjusted for age, sex.

Model 2: included variables in model 1 and further BMI, MAP, Hs-CRP, LDL-C, Diabetes mellitus, physical activity, educational level, smoking, drinking, antihypertensive agents and lipid-lowering drugs therapy.

### 3.5. Results of the stratification and sensitivity analyses

There were no significant interactions of age, sex for the association between non-HDL-C and baPWV and CVD. However, this result was invalidated in taking lipid-lowering medication populations, compared with the Normal both non-HDL-C and baPWV group, the HRs in each group were 1.43 (0.73,2.82), 1.10 (0.61,2.00), and 1.68 (0.92,3.05), respectively.

To exclude the effects of drugs and to verify their robustness, we repeated the multivariate Cox proportional risk regression analysis after the exclusion those participants who developed CVD the first year of the study, took anti-hypertensive, hypoglycemic, or lipid-lowering medications, and those with ABI ≤ 0.9. We found that the risk of CVD was higher in participants with both high non-HDL-C concentration and high baPWV, consistent with the results of the main outcome analysis, and as shown in Tables 4, 5.

**Table 4:** Stratified analysis of CVD according to non-HDL-C and baPWV.

	**Sex (*****P*** **for interaction 0.90)**		**Age (*****P*** **for interaction 0.07)**		**Lipid-lowering drugs (*****P*** **for interaction 0.41)**	
	**Men** **(*****N*** = **32,338)**	**Women** **(*****n*** = **12,713)**	**Age**<**45 years old** **(*****N*** = **17,597)**	**Age** ≥**45 years old** **(*****N*** = **27,454)**	**No** **(*****N*** = **2,927)**	**Yes** **(*****N*** = **42,124)**
Normal both non-HDL-C and baPWV	1.00	1.00	1.00	1.00	1.00	1.00
High non-HDL-C and normal baPWV	1.27 (0.93, 1.73)	1.04 (0.44, 2.43)	0.82 (0.19, 3.61)	1.28 (0.95, 1.73)	1.36 (1.03, 1.84)	1.43 (0.73, 2.82)
Normal non-HDL-C and high baPWV	1.56 (1.28, 1.89)	1.62 (0.82, 3.20)	2.42 (0.81, 7.24)	1.58 (1.31, 1.91)	1.62 (1.34, 1.97)	1.10 (0.61, 2.00)
High both non-HDL-C and baPWV	1.67 (1.27, 2.20)	1.65 (1.02, 2.67)	2.56 (1.03, 6.37)	1.67 (1.28, 2.17)	1.89 (1.47, 2.36)	1.68 (0.92, 3.05)

Model adjusted for age, sex, BMI, MAP, Hs-CRP, LDL-C, Diabetes mellitus, physical activity, educational level, smoking, drinking, antihypertensive agents and lipid-lowering drugs therapy.

**Table 5:** Sensitivity analysis of the influence of the combined effects of non-HDL-C and baPWV on CVD.

**Group**	**HR (95%CI)**		
	**Sensitivity analysis 1**	**Sensitivity analysis 2**	**Sensitivity analysis 3**
**Cardiovascular disease**			
High non-HDL-C and normal baPWV	1.00	1.00	1.00
Normal non-HDL-C and high baPWV	1.37 (1.02, 1.84)	1.35 (1.03, 1.77)	1.18 (0.72, 1.94)
High both non-HDL-C and baPWV	1.49 (1.20, 1.80)	1.58 (1.31, 1.90)	1.61 (1.18, 2.18)
High both non-HDL-C and baPWV	1.74 (1.35, 2.20)	1.90 (1.53, 2.37)	1.93 (1.32, 2.47)
**Myocardial infarction**			
Normal both non-HDL-C and baPWV	1.00	1.00	1.00
High non-HDL-C and normal baPWV	1.67 (0.92, 3.02)	2.20 (1.25, 3.84)	1.23 (0.54, 2.78)
Normal non-HDL-C and high baPWV	1.70 (1.07, 2.74)	1.60 (1.03, 2.49)	1.10 (0.61, 2.37)
High both non-HDL-C and baPWV	2.20 (1.20, 4.02)	2.33 (1.40, 3.86)	2.10 (0.86, 5.13)
**Ischemic stroke**			
Normal both non-HDL-C and baPWV	1.00	1.00	1.00
High non-HDL-C and normal baPWV	1.26 (0.90, 1.76)	1.23 (0.90, 1.68)	1.06 (0.59, 1.91)
Normal non-HDL-C and high baPWV	1.42 (1.13, 1.78)	1.56 (1.27, 1.91)	1.72 (1.22, 2.43)
High both non-HDL-C and baPWV	1.74 (1.33, 2.28)	1.83 (1.43, 2.33)	1.88 (1.27, 2.79)

Sensitivity analysis 1: Excluding outcome events within the first year of follow-up (n = 313).

Sensitivity analysis 2: Excluding participants with ABI < 0.9 (n = 967).

Sensitivity analysis 3: Excluding participants who underwent treatment with anti-hypertensive, hypoglycemic, or lipid-lowering medications (n = 13,288).

Sensitivity analysis 1 and 2: adjusted for age, sex, BMI, MAP, Hs-CRP, LDL-C, Diabetes mellitus, physical activity, educational level, smoking, drinking, antihypertensive agents and lipid-lowering drugs therapy.

Sensitivity analysis 3: adjusted for age, sex, BMI, MAP, Hs-CRP, LDL-C, Diabetes mellitus, physical activity, educational level, smoking, drinking.

## 4. Discussion

To the best of our knowledge, this is the largest longitudinal cohort study (45,051 participants, a 5.04-year follow-up period) conducted in China to explore the relationships of non-HDL-C concentration and baPWV with CVD. We found that both non-HDL-C and baPWV are positively associated with the risk of CVD. More importantly, a combination of high non-HDL-C concentration and high baPWV further increases the risk of CVD. In addition, taking lipid-lowering could remove those association.

In the present study, we found that relative to individuals with normal non-HDL-C or no arteriosclerosis, those with higher non-HDL-C concentration or arteriosclerosis tended to be at a higher risk of CVD. Moreover, participants with arteriosclerosis were at higher risk than those with a high non-HDL-C concentration. Previous studies have shown that the incidence and progression of arteriosclerosis are closely related to hypertension, smoking, obesity, and abnormal glucose and lipid metabolism. Not only abnormal lipid metabolism, but also a number of other factors, such as diabetes, hypertension, smoking, and obesity increase the risk of CVD (28–31). Therefore, compared to hypercholesterolemia, atherosclerosis has a larger impact on the incidence of CVD. The results of the present study are consistent with those of several previous large observational studies. Pencina et al. (18) showed that the risk of CVD in people with a high non-HDL-C concentration is three times higher than in people with a normal non-HDL-C concentration. In addition, in a meta-analysis of 14 Japanese cohort studies consisting of a total of 14,673 observations, Ohkuma et al. (17) found that the risk of CVD in the highest quintile group of baPWV is 3.50 times higher than that of the lowest quintile group. The results of these studies implied that both non-HDL-C and baPWV could be used as independent predictors of the risk of CVD.

In addition, we found that the highest risk of CVD was in individuals with both high non-HDL-C and high baPWV, and that this was 1.89 times higher than in individuals with both normal non-HDL-C concentration and baPWV. Although the present study is the first to demonstrate the combined effect of non-HDL-C and baPWV on the risk of CVD, similar analyses (32–34) of the combined effects of lipids and blood pressure on CVD risk have been performed previously. Yang et al. (34) showed that individuals with both hypertension and hypercholesterolemia are at a higher risk of CVD than those with hypertension or hypercholesterolemia alone in a study of 5,092 participants who were followed for 20.84 years.

Moreover, we repeated the analysis after the exclusion of outcome events within the first year of follow-up, participants with ABI < 0.9 and participants who underwent treatment with anti-hypertensive, hypoglycemic, or lipid-lowering medications. We found that the strength of association between non-HDL-C and IM or ischemic were weaker. The results are similar with those of several previous studies (35–38). Okamura et al. (35) showed that nor non-HDL-C cannot predict any subtype of stroke risk. Noda et al. (36) showed that higher concentrations of non-HDL-C were associated with an increased risk of mortality from coronary heart disease for men, but not for women. These inconsistent results may be caused by the differences in study design and method, participants' characteristics, and the covariates adjusted in the multivariate models (39).

The mechanism underpinning the relationship of a combination of arteriosclerosis and high non-HDL-C concentration with a higher risk of CVD remains poorly understood. On the basis of previous findings, we considered that it may involve the following. First, high non-HDL-C concentrations result in cholesterol accumulation in the arterial wall, which leads to atherosclerosis. Subsequently, the oxidative stress and chronic low-grade inflammation induced by the excessive lipid deposition stimulate leukocytes to release a variety of cytokines and adhesion molecules, causing them to adhere to the vascular endothelium and penetrate the intima, thereby increasing vascular resistance, leading to atherosclerosis and endothelial dysfunction, and ultimately increasing the risk of CVD (40–45).

Our study findings have important clinical implications. As we know, high baPWV and high non-HDL-C were positively associated with the risk of CVD independently. Our findings underscore that a higher risk of CVD in individuals with a combination of both high non-HDL-C and baPWV than in those with one of them alone. Furthermore, participants who underwent treatment with lipid-lowering medications could decrease the risk and remove those association between non-HDL-C and baPWV and CVD. Therefore, early identification of high non-HDL-C and high baPWV in the general population, should greatly assist the prevention of CVD, and lipid-lowering medications, such as statins are an effective treatment to reduce CVD for those individuals with high non-HDL-C or high baPWV.

There were several limitations to the present study. First, we assessed arterial stiffness using baPWV, rather than cfPWV, which is considered to be the gold standard method of evaluation. However, previous studies have shown a close correlation between baPWV and cfPWV (46). Second, the duration of follow-up was not very consistent among the participants, which may have influenced the findings. Third, most of the participants were men of a single ethnicity; therefore, the findings cannot be directly extrapolated to other ethnic groups. However, the Kailuan cohort comprises a large group with diverse occupations that has been closely followed; therefore, the present findings are likely to be representative and reliable. Four, we only gathered the all-cause mortality but not specific cause of death, which is the constraint on some outcomes to attain.

In summary, we have shown a higher risk of CVD in individuals with a combination of both high non-HDL-C and high baPWV than in those with one of them alone. Moreover, the closer relationship of high baPWV suggests a larger contribution to the risk. In addition, which taking lipid-lowering could remove those association between non-HDL-C and baPWV and CVD. Therefore, early identification of high non-HDL-C and high baPWV, and especially the measurement of baPWV in the general population, should greatly assist the prevention of CVD.

## Data availability statement

The original contributions presented in the study are included in the article/supplementary material, further inquiries can be directed to the corresponding authors.

## Ethics statement

The studies involving human participants were reviewed and approved by the Ethics Committee of the Kailuan General Hospital (Approval Number: 2006-05). The patients/participants provided their written informed consent to participate in this study.

## Author contributions

QZ, HW, XW, YL, WW, XY, and ZH designed the study idea. ZKC, ZFC, QL, HSZ, ML, and YZ analyzed and interpreted the data. QZ, YL, XW, QL, HSZ, YZ, KW, and HCZ drafted the manuscript. SW and YC reviewed the manuscript. All authors have read and approved the final manuscript.
